# Advances in Lipid Nanoparticles for mRNA-Based Cancer Immunotherapy

**DOI:** 10.3389/fchem.2020.589959

**Published:** 2020-10-23

**Authors:** Maria L. Guevara, Francesca Persano, Stefano Persano

**Affiliations:** ^1^Barts Cancer Institute, Queen Mary University of London, London, United Kingdom; ^2^Department Matematica e Fisica 'Ennio De Giorgi', Università del Salento, Lecce, Italy; ^3^Nanomaterials for Biomedical Applications, Istituto Italiano di Tecnologia (IIT), Genova, Italy

**Keywords:** monoclonal antibodies, CAR T cells, cancer vaccines, lipid nanoparticles, therapeutic mRNA, cancer immunotherapy

## Abstract

Over the past decade, messenger RNA (mRNA) has emerged as potent and flexible platform for the development of novel effective cancer immunotherapies. Advances in non-viral gene delivery technologies, especially the tremendous progress in lipid nanoparticles' manufacturing, have made possible the implementation of mRNA-based antitumor treatments. Several mRNA-based immunotherapies have demonstrated antitumor effect in preclinical and clinical studies, and marked successes have been achieved most notably by its implementation in therapeutic vaccines, cytokines therapies, checkpoint blockade and chimeric antigen receptor (CAR) cell therapy. In this review, we summarize recent advances in the development of lipid nanoparticles for mRNA-based immunotherapies and their applications in cancer treatment. Finally, we also highlight the variety of immunotherapeutic approaches through mRNA delivery and discuss the main factors affecting transfection efficiency and tropism of mRNA-loaded lipid nanoparticles *in vivo*.

## Introduction

mRNA-based therapeutics have emerged as a highly appealing new class of drugs, revolutionizing cancer immunotherapy by finding application in different types of anticancer approaches, such as therapeutic vaccines, monoclonal antibodies, immunomodulatory drugs and CAR cell therapies (Van Lint et al., 2012; Kranz et al., 2016; Pardi et al., 2018; Foster et al., 2019; Hoecke and Roose, 2019). In comparison to other functional biomolecules such as plasmid DNA (pDNA) and recombinant proteins, mRNA exhibits several therapeutic benefits, thereby rendering it highly desirable for the development of a new generation of cancer immunotherapy drugs (Pastor et al., 2018).

Firstly, mRNA possesses a superior safety profile indeed, unlike pDNA, mRNA cannot integrate into the genome and thus avoids potential insertional mutagenesis (Sahin et al., 2014). Moreover, mRNA efficiently transfects both mitotic and non-mitotic cells, as it does not require to enter into the nucleus since it exerts its function in the cytoplasmic compartment (Sahin et al., 2014).

Translatability and stability of mRNA as well as its immunostimulatory activity are further intrinsic features that make it the most attractive type of therapeutic agent emerged in the last decade, especially for cancer immunotherapy (Pastor et al., 2018; Guevara et al., 2019b).

Finally, large-scale production of Good Manufacturing Practice (GMP)-grade mRNA is relatively simple, fast and inexpensive, since mRNA is synthesized in a cell-free system, and its manufacturing can be obtained in standardized and controlled conditions (Sahin et al., 2014; Pardi et al., 2018; Guevara et al., 2019b).

Despite the enormous potential of mRNA-based therapies, only recently has their therapeutic application become possible, as a consequence of the considerable progresses of nanomedicine in the design of non-viral vectors for gene delivery (Guan and Rosenecker, 2017; Kaczmarek et al., 2017). In this regard, various type of nanoparticles have been investigated as mRNA delivery systems, but lipid nanoparticles have been the most extensively explored for mRNA-based immunotherapy, enabling a variety of new antitumor treatments currently in preclinical development and some undergoing clinical trials (Gómez-Aguado et al., 2020).

The use of lipid-based nanocarriers has addressed key issues for mRNA transfection into target cells by improving its protection from degradation in the extracellular compartments, as well as by facilitating cellular uptake and delivery to an appropriate intracellular compartment (Wadhwa et al., 2020). Herein, we will provide an overview on the recent advances in the field of lipid-based nanoparticles and on the design of mRNA delivery platforms for various forms of cancer immunotherapy.

## Lipid-Based Nanoparticles for mRNA Delivery: Basic Formulation and Structural Organization

The encapsulation of mRNA into a carrier is essential to fully harness its therapeutic power by ensuring protection from extracellular RNase degradation and simultaneously promoting cellular uptake and endosomal escape of mRNA (Guan and Rosenecker, 2017; Guevara et al., 2019b).

To enable mRNA encapsulation, protection, and transfection, amine-containing nanomaterials are commonly used as non-viral platforms (Kranz et al., 2016; McKinlay et al., 2017; Oberli et al., 2017; Persano et al., 2017; Zhang et al., 2019). Lipid-base formulations represent the most developed tool for mRNA delivery (Wadhwa et al., 2020).

**Figure 1:**
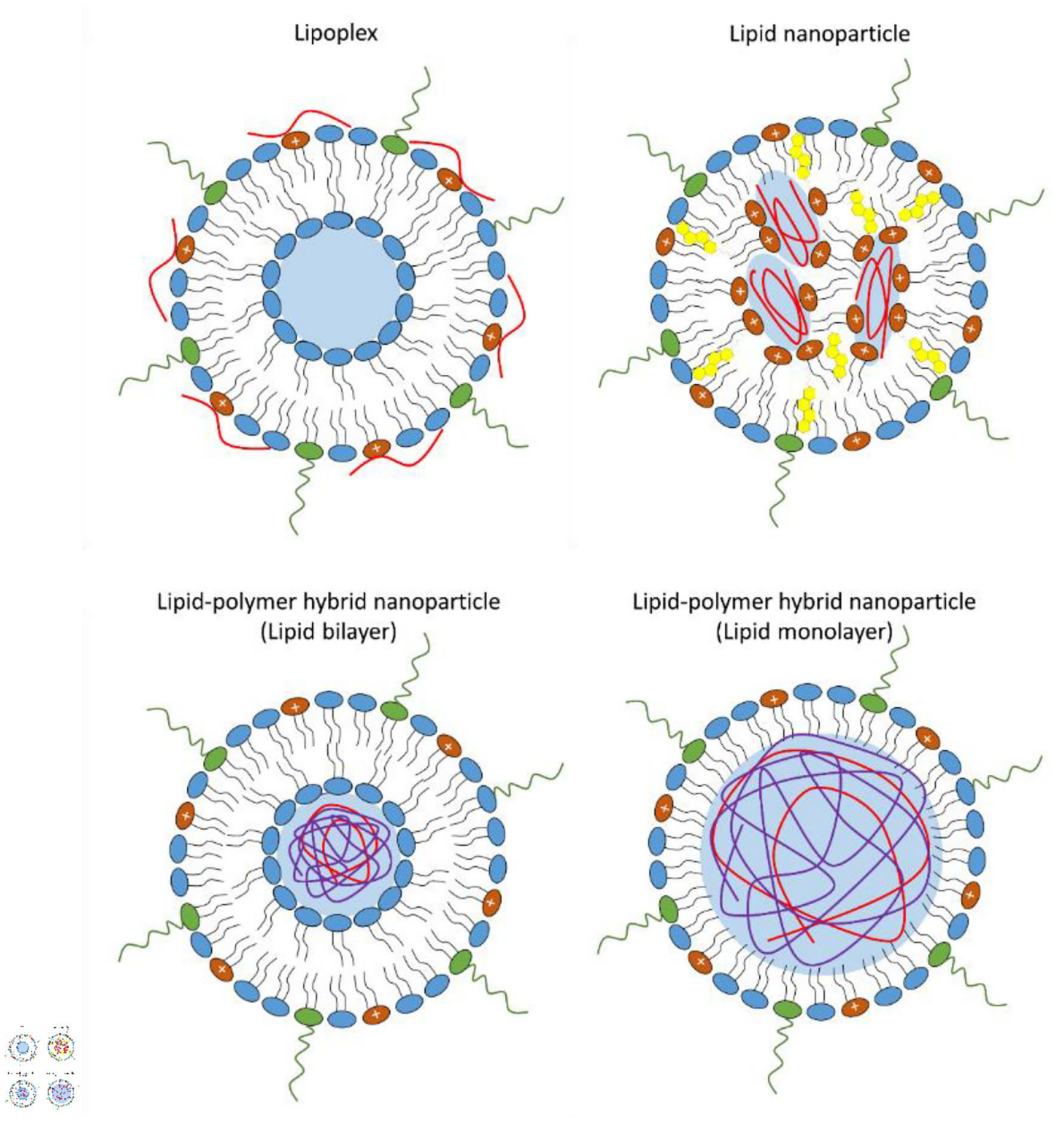
Schematic representation of the different types of lipid-base nanovectors: lipoplex, lipid nanoparticle, lipid-polymer hybrid nanoparticle where the lipid shell can be organized as a bilayer or monolayer.

Lipoplexes, consisting of cationic liposomes interacting electrostatically with the negative charges of the phosphate backbone of mRNA, were the earliest lipid-based delivery systems successfully employed to introduce mRNA molecules into target cells (Figure 1) (Felgner and Ringold, 1989; Kranz et al., 2016). However, after a first brief phase of great enthusiasm, lipoplexes have shown important concerns, such as high instability, relatively low transfection efficiency and poor customizable composition, given that they are often formulated with an excess of cationic charges not only to promote mRNA binding but also to facilitate the interaction with the anionic phospholipids in the plasma membrane and subsequently promote its uptake by endocytosis (Li and Huang, 1997; Xue et al., 2015; Guevara et al., 2019b; Wahane et al., 2020).

These drawbacks have limited further application of nucleic acid-loaded lipoplexes, thus shifting the current interest on lipid nanoparticles, which have demonstrated superior stability, structural plasticity and enhanced gene delivery (Xue et al., 2015; Guevara et al., 2019b).

A typical lipid nanoparticle formulation is composed of pH-responsive lipids or cationic lipids bearing tertiary or quaternary amines to encapsulate the polyanionic mRNA; neutral helper lipids such as zwitterionic lipid [i.e., 1,2-dioleoyl-sn-glycero-3-phosphoethanolamine (DOPE) or 1,2-distearoyl-sn-glycero-3-phosphocholine (DSPC)] and/or sterol lipid (i.e., cholesterol) to stabilize the lipid bilayer of the lipid nanoparticle and to enhance mRNA delivery efficiency; and a polyethylene glycol (PEG)-lipid to improve the colloidal stability in biological environments by reducing aspecific absorption of plasma proteins and forming a hydration layer over the nanoparticles (Cullis and Hope, 2017; Guevara et al., 2019b). The morphology of lipid nanoparticles is not like a traditional liposome, characterized by a lipid bilayer surrounding an aqueous core, indeed, they possess an electron-dense core, where the cationic/ionizable lipids are organized into inverted micelles around the encapsulated mRNA molecules (Figure 1) (Cullis and Hope, 2017; Guevara et al., 2019b).

Recently, lipid-polymer hybrid nanoparticles have emerged as novel mRNA delivery systems combining the advantages of biodegradable polymeric nanoparticles and liposomes (Persano et al., 2017; Islam et al., 2018; Guevara et al., 2019b). Lipid-polymer hybrid nanoparticles consist of a biodegradable mRNA-loaded polymer core coated with a lipid layer (Persano et al., 2017; Islam et al., 2018; Guevara et al., 2019b). Usually, the lipid envelope is organized into a lipid bilayer or lipid monolayer containing a mixture of cationic or ionizable lipids, helper lipids, and pegylated lipid (Figure 1).

Lipid-polymer hybrid nanoparticles, thanks to their structural design, can offer a series of benefits such as small size, high nucleic acid condensation efficiency, large functionalizable surface that can be easily modified by the binding of different functional groups, and prolonged blood circulation time (Guevara et al., 2019b). In addition, the specific physicochemical properties of hybrid lipid-polymer nanostructures can potentially result in a different interaction of the delivered mRNA with innate RNA sensors, consequently altering the immunogenicity and safety profile of lipopolyplex-based immunotherapies (Van der Jeught et al., 2018).

The above benefits of hybrid lipid-polymer formulations have been highlighted in a recent study were an mRNA-loaded lipid-polymer platform functionalized with mannose receptor targeting moieties to promote dendritic cell (DC) targeting *in vivo* was employed (Van der Jeught et al., 2018). The formulation exhibited excellent hemocompatibility and the expression of the mRNA cargo was preferentially restricted to splenic antigen presenting cells (APCs) upon systemic administration. Furthermore, vaccination with the lipopolyplex formulation elicited a potent T-cell-mediated immune response and manifested superior effectiveness in inhibiting tumor growth compared to intravenous immunization with a lipoplex-based mRNA vaccine (Van der Jeught et al., 2018). Early innate responses to hybrid lipid-polymer vaccine formulation were characterized by a type I interferon (IFN) response in the spleen. Nevertheless, unlike conventional lipoplexes, the hybrid lipid-polymer nanovaccine did not rely on type I IFN responses to generate cytotoxic T-cell effectors (Van der Jeught et al., 2018). This unlooked behavior of lipopolyplex nanostructures could enable the preparation of new anticancer therapeutic vaccines with a more moderate pro-inflammatory profile, but with an equal capacity to promote a potent immune response, representing a valid alternative to the lipid formulated mRNA vaccines currently under investigation in early phase clinical trials.

## Cellular Internalization and Endosomal Escape of mRNA-Loaded Lipid-Based Nanoparticles

Although the mechanism that leads to the internalization of RNA-loaded lipid-based nanoparticles has not been fully clarified, experimental insights revealed that the process involves clathrin-dependent endocytosis followed by micropinocytosis, that is the major uptake mechanism (Gilleron et al., 2013; Wang and Huang, 2013). The initial interaction of nanoparticles with the cell plasma membrane of the target cells can be promoted or accelerated by the presence of positive charges or active targeting ligands on the outer surface, which can interact with the negatively charged cell membrane components or with specific proteins exposed at the cell membrane of the target cell (Hajj and Whitehead, 2017).

Once the lipid nanoparticles are engulfed into the cell, they follow the conventional endocytic route, trafficking first into early endosomes, then into late endosomes, and finally into lysosomes where the RNA is enzymatically degraded. It has been estimated that only a small fraction (1–2%) of lipid nanoparticles can evade the endosomal pathway before they reach the lysosomes and this tend to vary between cell types (Gilleron et al., 2013). The proton sponge effect was initially considered the dominant mechanism leading to the endosomal escape of the RNA-loaded lipid nanoparticles. However, increasing evidence indicates that the endosomal escape mechanism of lipid nanoparticles is much more complex, and involves the docking of the lipid nanoparticles at the endosomal membrane, triggering membrane fusion and destabilization of the endosomal lipid bilayer, with consequent release of the genetic cargo into the cytosol (Zelphati and Szoka, 1996; Gilleron et al., 2013).

Previous studies revealed that endosomal escape occurs mainly from early endosomes or macropinosomes before their fusion with lysosomes, since late endosomes and lysosomes are characterized by lower leakiness, due to the variation in the lipid composition occurring during endosome maturation (Gilleron et al., 2013; Wang and Huang, 2013). These changes in the cell membrane lipid composition consist in a decrease of the cholesterol content, the hydrolysis of sphingomyelin and increased levels of phosphatidylcholine in the membranes of late endosomes and lysosomes. However, a recent study suggested that late endosome/lysosome formation could be essential for the functional delivery of mRNA (Patel et al., 2017). Indeed, Rab7A-deficient cells exhibited not significant changes in mRNA-uptake but a strong decrease in the transfection efficiency compared to wild-type cells. Conversely, the absence of Rab4A or Rab5A, both localized at the early/recycling endosomes, had limited effects on cell transfection efficiency. Interestingly, the authors showed that mRNA electroporation of Rab7A knockout cells was not able to rescue the basal transfection efficiency obtained in wild-type cells, and provided evidence that the late endosome/lysosome structure can positively control the translation of the delivered mRNA by serving as hub for the mammalian target of rapamycin complex 1 (mTORC1)-mediated signaling pathway (Patel et al., 2017).

Recently, Maugeri et al. showed that after endocytosis a small fraction of mRNA-loaded lipid nanoparticles can immediately evade the endocytic route and be consigned to the recycling pathway to be expelled by exocytosis (Maugeri et al., 2019). After secretion, the mRNA packed into extracellular vesicles can be transferred in other cells *in vitro* and blood/organs *in vivo* and produce new copies of protein (Maugeri et al., 2019).

All these findings suggest that mRNA transfection mediated by lipid nanoparticles is an overly complex process, strongly influenced by several factors, such as uptake mechanism, endosomal maturation, endosomal recycling, and may vary widely between different cell types. Inefficient endosomal escape efficacy and precise tissue/cell targeting efficiency remain the major challenges for mRNA delivery by lipid-based nanoplatforms. A better understanding of the mechanisms regulating the biodistribution, internalization and endosomal escape of mRNA-loaded lipid nanoparticles will help in the development of next generation nanoparticle-based mRNA immunotherapies with increase efficacy, safety, and clinical translatability.

## Lipid Composition of Lipid-Based Nanoparticles for mRNA Delivery

### Cationic Lipids

**Figure 2:**
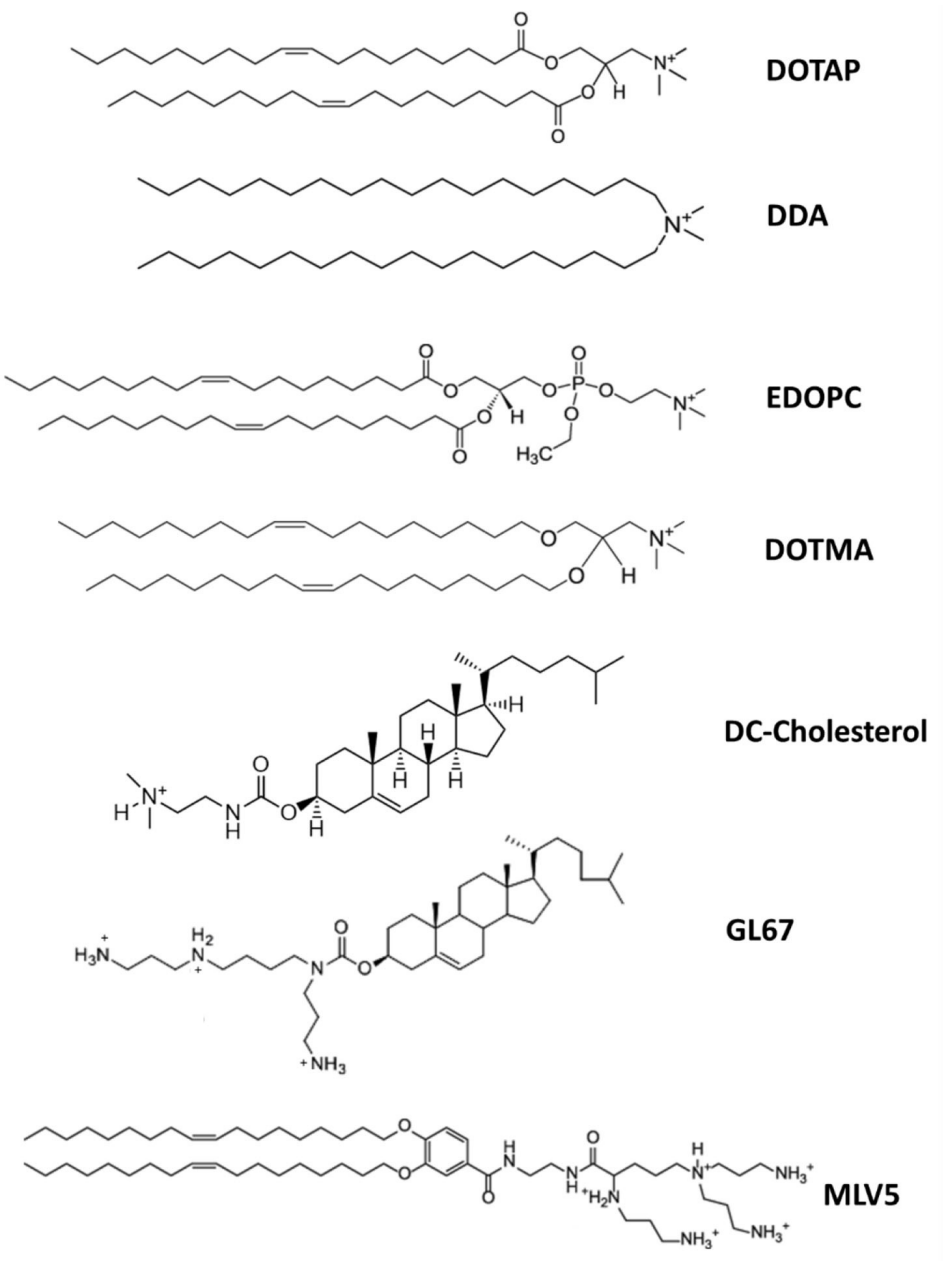
Chemical structure of the major cationic lipids utilized for mRNA delivery.

Cationic lipids are amphiphilic molecules, consisting of a positively charged polar head group, and a hydrophobic tail domain, that in aqueous solution spontaneously self-assemble into higher order aggregates (Figure 2) (Guevara et al., 2019b). Thanks to their cationic amino groups, they can electrostatically interact with the negatively charged phosphate groups of mRNA molecules and allow their entrapment in a lipid-based nanoparticle (Guevara et al., 2019b).

The use of delivery systems based on permanently cationic lipids have proven to be effective for mRNA *in vitro* transfection and was described for the first time more than 30 years ago (Felgner and Ringold, 1989; Malone et al., 1989). In this study, a relative high transfection efficiency was achieved using a lipoplex structure, obtained by complexing mRNA with a liposome containing the synthetic cationic lipid, N-[1-(2,3-dioleyloxy)propyl-N,N,N-trimethylammonium chloride (DOTMA), and the helper lipid DOPE (Malone et al., 1989). After this encouraging early result, further mRNA therapeutic development was abandoned, due to its high fragility and the inadequate knowledge at that time on the potential of non-viral vectors in protecting and efficiently deliver RNA molecules into eukaryotic cells (Guevara et al., 2019b). The recent revival interest in the use of mRNA-based immunotherapies has encouraged an advancement on the design of cationic lipid-based nanocarriers mostly for cancer immunotherapy (Guevara et al., 2019b).

On this regard, the study from 2016 by Kranz et al. has represented the culmination of several years of interdisciplinary research on mRNA-based drug optimization and has provided the bases for the development of novel mRNA-based cancer immunotherapies (Kranz et al., 2016). The authors showed that mRNA-lipoplexes composed of DOTMA/DOPE or 1,2-dioleoyl-3-trimethylammonium-propane (DOTAP)/DOPE lipids, formulated by gradually decreasing their surface charge from positive to negative, were able to protect antigen-encoding mRNA from extracellular ribonucleases, efficiently accumulating in the spleen and delivering the mRNA into DCs upon systemic administration, with the consequent induction of an antigen-specific immune response (Kranz et al., 2016).

More recently, Cheng et al. reported the percentage of permanently cationic lipid contained in the formulation as the main factor affecting bio-distribution of pH-independent cationic lipid nanoparticles (Cheng et al., 2020). Surprisingly, by increasing the percentage of DOTAP lipid from 5 to 100%, the expression of the encapsulated luciferase-encoding mRNA shifted progressively from liver to spleen, and then to lung, demonstrating that the percentage of cationic lipid can be opportunately tailored for tissue-specific delivery via an intravenous administration route (Cheng et al., 2020).

Cationic lipid-based nanoparticles have seen a widespread use in the delivery of therapeutic mRNA, not only for their ability to form stable complexes with nucleic acids, but also because they have revealed intrinsic immunogenic properties attributable to the interaction with innate immune components, thus serving as immune adjuvants to enhance the immunogenicity of formulations.

For instance, the immunogenicity of the cationic lipid dimethyldioctadecylammonium (DDA) was illustrated already more than 50 years ago by Gall (Gall, 1966). DDA can act as a vaccine adjuvant, enhancing both cell-mediated and humoral immunity, and it has shown to be effective in different vaccine platforms, including mRNA-based vaccines (Henriksen-Lacey et al., 2010; Blakney et al., 2019). The adjuvant activity of DDA has been attributed to its positive surface charge and its ability to interact and stabilize antigens by ionic interactions. This was demonstrated by using fluorescently labeled ovalbumin (OVA) as a model antigen (Korsholm et al., 2007). The adsorption of the antigen onto DDA liposomes enhanced its uptake by APCs, in addition to increasing its immunogenicity as confirmed by the significant upregulation in the expression of maturation markers of APCs, all this resulted in the improvement of their effectiveness in antigen presentation (Korsholm et al., 2007). Similarly, both DOTAP- and DOTMA- based nanostructures have been reported to induce the activation of TLRs and NLRP3 inflammasome pathways (Lonez et al., 2014). Therefore, delivery platforms containing permanently cationic lipids can be opportunely designed and tuned to obtain novel mRNA-based immunotherapies with superior immunogenicity and therapeutic efficacy.

### Ionizable Lipids and Lipid-Like Polymers

**Figure 3:**
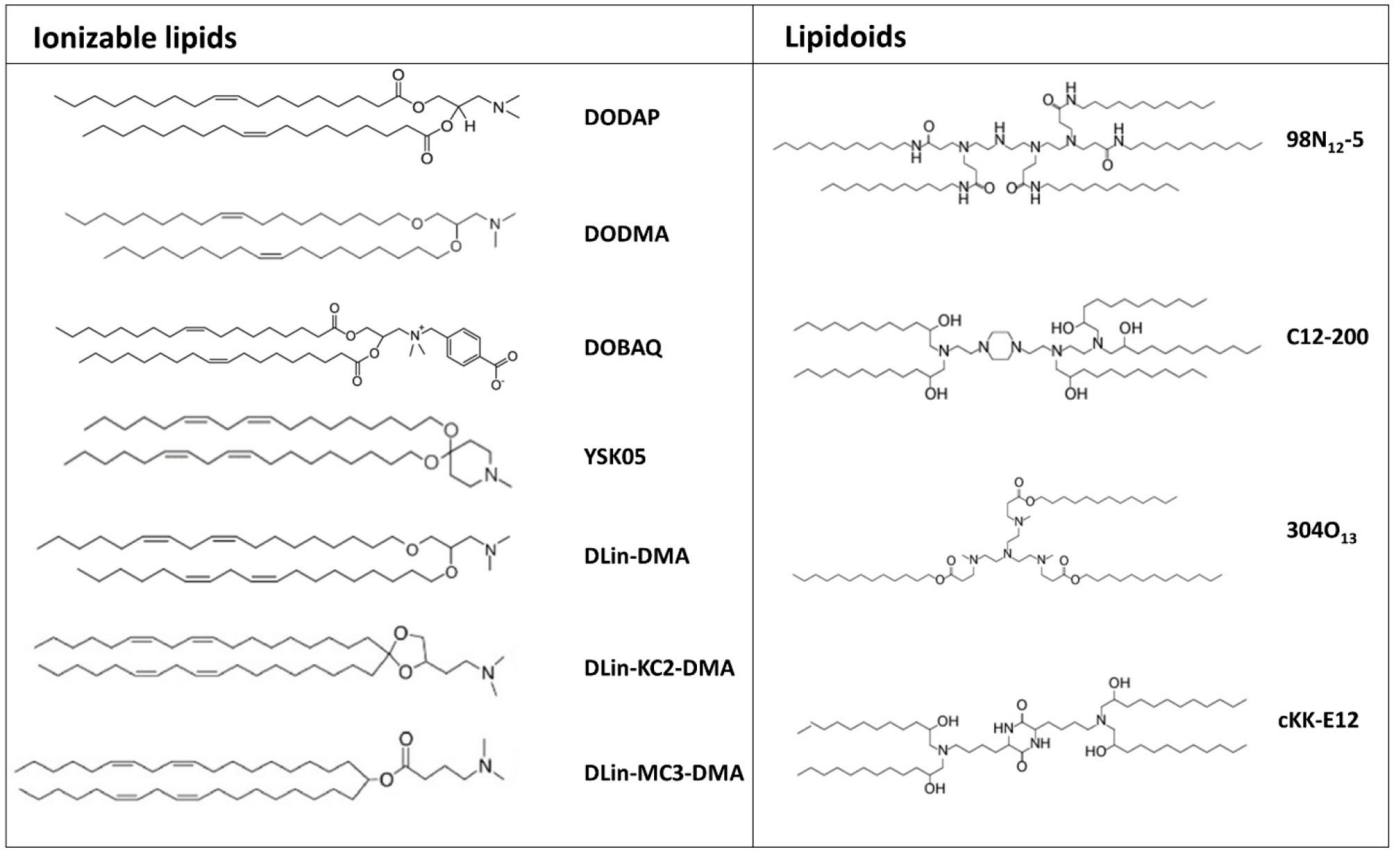
Chemical structure of the most common ionizable lipids and lipidoids used for mRNA delivery.

A second generation of transfecting lipids was developed due to the necessity of novel delivery systems for siRNA molecules with improved safety profile and able to accumulate more efficiently at the target site, avoiding sequestration by blood-filtering organs like liver and spleen, a phenomenal frequently observed mostly with positively charged nanoparticles (Figure 3) (Blanco et al., 2015).

In the first half of the 1990s, Cullis developed the first pH-responsive cationic lipid, bearing the unique feature that its net charge changed in response to the pH of its surroundings, acquiring a net positive charge in an acidic pH and maintaining a neutral charge in a physiological pH (Bailey and Cullis, 1994). Thus, mRNA encapsulation into pH-responsive lipid nanoparticles is achieve only in acidic conditions. Nanoparticles formulated with these lipids have a minimal positive charge density in the bloodstream, and therefore tend to display a superior biocompatibility and a reduced off-target accumulation (Tam et al., 2013). Several ionizable lipids have been proposed, initially for siRNA delivery and, recently, their application has been extended to mRNA delivery (Figure 3).

The initial difficulties encountered in achieving efficient mRNA delivery by using lipid formulations designed for siRNA delivery pointed out that delivery systems should be specifically tailored for mRNA, as it has different features compared to siRNA. Therefore, previously proposed lipid formulations have been re-optimized and new ionizable synthetic lipids have been introduced with the aim of enhancing the delivery and translation of mRNA *in vivo*, and thus improving its therapeutic effect.

Several studies conducted in this area have allowed to identify the pKa value as the dominant factor affecting the transfection efficiency of ionizable lipids, with an optimal pKa range of 6.2–6.5 (Cullis and Hope, 2017). DLin-MC3-DMA (MC3), having an optimized pKa value of 6.44, represents one of the most powerful pH-dependent cationic lipids that has been synthesized for RNA delivery, and it has been successfully employed for protein replacement therapy of genetic diseases such as the neurodegenerative disease Friedreich's ataxia (Tam et al., 2013; Nabhan et al., 2016; Arteta et al., 2018). MC3-based lipid nanoparticles encapsulating either luciferase or farataxin encoding mRNA and intrathecally injected in mice resulted in high protein expression into dorsal root ganglion neurons (Nabhan et al., 2016).

Lipid-like materials also known as lipidoids, represent a new generation of ionizable lipids characterized by protonable tertiary-amino groups and highly hydrophobic side chains, which play a significant role in determining transfection efficiency (Figure 3). Indeed, experimental observations helped to clarify the huge impact that the chemical structure of the tail portion of cationic lipids could have on the transfection efficiency of lipid-based nanoparticles. In this regard, a recently conducted screening study revealed that longer and unsaturated alkyl tails could enhance mRNA delivery efficiency (Fenton et al., 2016).

Similarly, a biodegradable ionizable lipid obtained by modifying the hydrophobic tail of MC3 lipid through the introduction of ester and alkyne groups, in addition to showing an improved tolerability, displayed an enhanced transfection efficiency. In particular, Miao et al. observed that the incorporation of an unsaturated alkyne group, rather than double bounds, in the non-polar tail of the ionizable lipid can improve the fusogenicity with the endosomal membrane, and consequently facilitate endosomal escape and mRNA release into the cytosol (Miao et al., 2020). Additionally, its co-formulation with cKK-E12 pH-responsive cationic lipid synergistically boosted mRNA delivery into hepatocytes, offering early evidence that novel and more efficient delivery systems could be potentially obtained from the co-formulation of distinct ionizable lipids. cKK-E12-based lipids have been shown to enhance the serum stability and protein binding of the particles (Miao et al., 2020).

Ionizable lipid-based nanoparticles were recently utilized to facilitate the development of several mRNA-based immunotherapies for cancer treatment. For instance, Oberli et al. demonstrated the efficacy of ionizable lipid-based nanoparticles for antigen encoding mRNA-based anti-tumor vaccination (Oberli et al., 2017). Interestingly, the authors reported that unmodified mRNA led to a significantly higher number of antigen-specific CD8^+^ T cells in peripheral blood, compare to modified mRNA, upon subcutaneous administration. Finally, the anti-tumor vaccine obtained by loading a TRP-2 encoding mRNA into ckk-E12-based lipid nanoparticles significantly suppressed tumor growth and extended the survival of B16 F10 tumor-bearing mice, and the addition of LPS into the formulation further improved this effect (Oberli et al., 2017). A similar formulation was proposed in a recently published study by Stadler et al. showing that antibody-encoding mRNA delivery can enable antibody-mediated cancer immunotherapy (Stadler et al., 2017). Systemically-administered modified-mRNA encoding for a bispecific antibody directed against the T cell receptor (TCR)–associated CD3 complex and a tumor-associated antigen loaded into a hybrid polymer/lipid-based formulation was shown to significantly impair tumor growth in a murine tumor model (Stadler et al., 2017).

### Helper Lipids and Stealth Lipids

**Figure 4:**
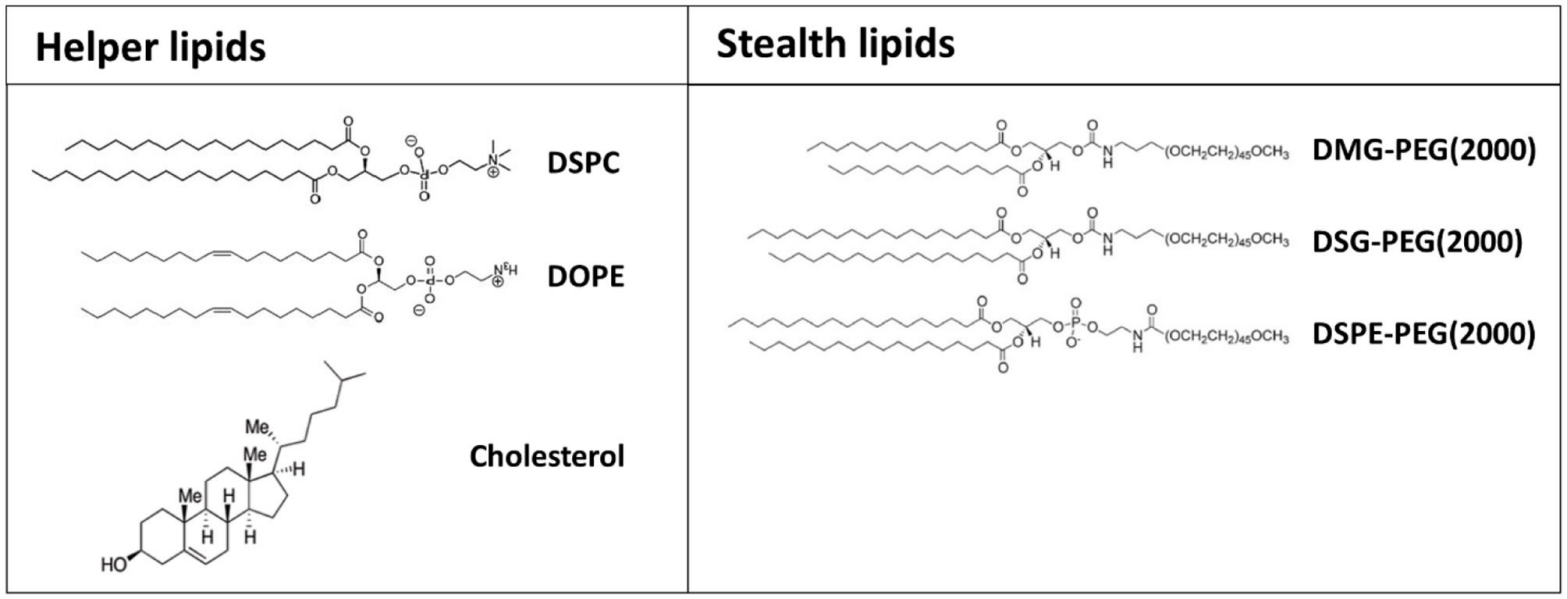
Chemical structure of the most common helper and stealth lipids employed for the preparation of formulated mRNA.

In addition to charged or ionizable materials, lipid-based nanoformulations typically comprise supplementary components including cholesterol, for improving nanoparticle's stability; helper lipid, such as DSPC and DOPE, to facilitate the maintenance of the lipid bilayer structure and to promote endosomal release; and a PEG-conjugated lipids to prevent opsonization by serum proteins, thus enhancing the circulation time of nanoparticles (Figure 4) (Guevara et al., 2019b).

The ratio between the different types of lipids in the formulation of nanoparticles can substantially affect their efficacy as mRNA delivery systems (Oberli et al., 2017). Compared with lipid-based nanoparticles developed for achieving efficient siRNA delivery, a vector optimized for mRNA encapsulation and release generally requires a reduced amount of ionizable cationic lipid and cholesterol, and higher amounts of helper lipid and PEG-lipid (Weng et al., 2020).

The addition of cholesterol into the formulation has been shown to the enhance transfection efficiency of lipid-base nanoparticles, potentially by promoting membrane fusion and endosomal escape and, as expected, the percentage of cholesterol has a considerable influence on intracellular gene delivery (Pozzi et al., 2012). Patel et al. reported that the inclusion of a naturally occurring cholesterol analogous (C-24 alkyl phytosterols) into lipid-based nanoformulations enhances mRNA delivery (Patel et al., 2020). In this regard, the length of the alkyl tail, the flexibility of sterol ring and the polarity associated with the hydroxyl group were found essential to maintain a high transfection efficiency. Interestingly, the structural examination of lipid nanoparticles containing phytosterols revealed a polymorphic shape and various degrees of multilamellarity, polymorphism and lipid partitioning (Eygeris et al., 2020). The modification of the tail with methyl and ethyl groups led to an increase of multilamellarity (>50% increase compared to cholesterol), whereas the addition of a double bond promoted lipid partitioning (>90% increase compared to cholesterol) (Eygeris et al., 2020). Lipid nanoparticles displaying multilamellar and polymorphic structures showed higher gene transfection.

Previous studies showed that by replacing DSPC, a helper lipid commonly included in siRNA lipid-based formulations, with DOPE, the mRNA delivery efficacy is strongly enhanced (Ball et al., 2018). This may be because phosphocholine-containing lipids usually inhibit membrane fusion-mediated endosomal escape, while unsaturated lipids, such as DOPE, can undergo a conformational change from a stable lamellar phase to an unstable hexagonal phase leading to membrane fusion (Harper et al., 2001; Sato et al., 2020).

However, Sato et al. reported that lipid nanoparticles formulated with a combination of a pH-sensitive cationic lipid with a hydrophobic tail longer than C18 and egg sphingomyelin (ESM), a phosphocholine-containing phospholipid, exhibited a dose-dependent transfection efficiency, while lipid nanoparticles with a classical scaffold length (=C18) failed in transfecting cells *in vitro*, demonstrating that the inhibitory effect of phosphocholine lipids on endosomal escape can be overcome by modifying the structure of the hydrophobic scaffold (Sato et al., 2020).

In order to prevent aggregation and favor a prolonged circulation time of nanoparticles in the bloodstream upon their systemic administration the coating of nanoparticles with a PEG-lipid through a process known as “PEGylation” is a commonly employed strategy. PEGylated nanoparticles are often referred as “stealth” nanoparticles, due their ability to avoid opsonization by serum proteins and detection by the reticuloendothelial system (RES) (Li and Huang, 2009).

The selection of the appropriate PEG-lipid is a crucial step in the design of mRNA delivery platforms and can have a huge impact on the carrier activity by shaping its pharmacokinetics. It has been demonstrated that the lipid anchor length determines how long the PEG-lipid remains incorporated in the lipid shell. PEG-lipids with longer anchors are stably included on the surface of the lipid layer and require more time to dissociate from it, thus preventing undesired interactions with proteins and cells, and consequently prolonging nanoparticles' blood circulation time (Zhu et al., 2017). On the other hand, PEGylation with PEG-lipids with longer anchors can negatively affect the uptake efficiency of nanoparticles by inhibiting their interaction with the plasma membrane of the target cells (Zhu et al., 2017). Moreover, PEGylated nanoparticles can be rapidly clear from the circulation upon secondary exposure, as consequence of antibody-mediated immune responses against the PEG component (Judge et al., 2006). Therefore, the use of PEG-lipids with shorter anchors, such as PEGylated 1,2-dimyristoyl-sn-glycerol (PEG-DMG, a C14-based lipid), which are gradually released from the surface of nanoparticles, appears to be an extremely successful approach to achieve high colloidal stability and cargo delivery into target cells (Tam et al., 2013). The reason for this is that the PEG-lipid is embedded into the lipid layer by hydrophobic interactions; hence, spontaneous de-PEGylation is a process that can be partially controlled changing their hydrophobic properties (Zhu et al., 2017).

PEG-lipids have been also extensively exploited to facilitate lipid-based nanoparticles' functionalization with specific targeting ligands that can promote their precise accumulation at the target site. For instance, to target DCs *in vivo*, mannose, which binds with high affinity to the Lectin Receptor DC-SIGN exposed on the surface of DCs, can be introduce in the formulation (Wang et al., 2018). Likewise, intravenously administrated anti-PECAM-1 antibody conjugated lipid nanoparticles have been successfully employed to achieve higher protein expression in the lungs compared to non-targeted counterparts (Parhiz et al., 2018).

Lipidic immune adjuvants have emerged as novel class of lipids that have been introduced into lipid-based mRNA formulations to further increase the immunogenicity of mRNA-based immunotherapies and “guide” the immune responses (Verbeke et al., 2017; Guevara et al., 2019a). In this regard, the Anderson group has developed multifunctional ionizable lipid-like materials capable of simultaneously facilitate mRNA delivery *in vivo* and act as immune adjuvants to potentiate anti-tumor immunity by promoting the activation of the stimulator of IFN genes (STING) pathway (Miao et al., 2019).

Taken together, these findings corroborate the importance of helper lipids and PEG-lipids in determining the fate and efficacy of lipid-based nanoparticles carrying mRNA, and emphasize the need for a deeper understanding of the relationship between the structural properties of lipid-based formulations and their endosomal escape activity and immunogenicity, thus enabling the design of high performing mRNA-based immunotherapeutics.

### Lipid-Based Nanoparticles' Preparation Techniques

**Figure 5:**
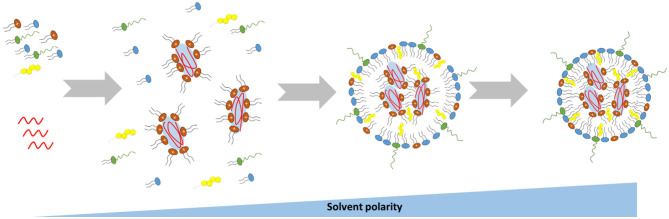
Schematic representation of the mechanism driving the self-assembly of mRNA-loaded lipid-based nanoparticles.

The method used for the preparation of lipid-based nanoparticles has been shown to be critical at determining the efficacy of lipid-based nanoformulations, as it has a direct impact on both their size and encapsulation efficiency (Cullis and Hope, 2017). Lipid-based nanoparticles are usually formed by ethanol injection nanoprecipitation technique, where the desired lipids dissolved in ethanol at an appropriate ratio, and the mRNA dissolved in an acidic aqueous buffer, are mixed together (Reichmuth et al., 2016; Cullis and Hope, 2017). The acidic pH is necessary to ensure the protonation of ionizable lipids (cationic lipids have a head group with a permanent positive charge) so that, after mixing of the two solutions, electrostatic interactions drive the formation of inverted micelles containing the mRNA surrounded by cationic lipids (Figure 5) (Cullis and Hope, 2017). The fast increase of solution's polarity promotes the aggregation of the inverted micelles, which is followed by the deposition of the other lipids on the surface of the nascent lipid-based nanoparticles (Figure 5) (Cullis and Hope, 2017). The PEG-lipid, that is the most hydrophilic lipid in the mixture, would be the last component to self-associate on the particle's surface to form an outer shell that stabilizes the nanoparticles (Cullis and Hope, 2017). Following the mixing step, nanoparticles are dialyzed against an aqueous buffer in order to increase the pH to a physiological value (Cullis and Hope, 2017).

Based on this method, the size of the resulting nanoparticles would be strongly influenced by the rate at which the polarity of the ethanol solution changes, which in turn is influenced by the mixing rate and the volumetric ratio between the aqueous and lipid phases. Therefore, a rapid mixing of the ethanol–lipid phase with excess water is essential for the preparation of uniform and small lipid-based nanoparticles (Cullis and Hope, 2017). In some cases the nanoprecipitation technique has been performed by replacing the ethanol with a different organic solvent, such as tert-butanol (t-But) or acetonitrile, with a consequent size reduction and improved polydispersity index of the nanoparticles (Matsui et al., 2015; Islam et al., 2018). Further factors that may significantly influence the size of the synthesized nanoparticles are the ratio of “core” lipid (cationic or ionizable lipid) to “surface” lipid in the lipid mix, the amount of PEGylated lipid contained in the formulation and the lipid composition (Cullis and Hope, 2017).

Nanoprecipitation technique has been successfully applied not only for the preparation of lipid nanoparticles but also for the synthesis of mRNA-loaded hybrid polymer-lipid nanoparticles (Kaczmarek et al., 2016).

New approaches for the synthesis of lipid-based nanoparticles directly mix the organic phase, containing the lipids, with the mRNA dispersed in the aqueous phase using a fluidic device (Cullis and Hope, 2017). The advantage of this strategy is that the flow and hence, the mixing rates, can be easily controlled through pumps. In this way, it has been possible to obtain nanoparticles with a diameter up to 70 nm and high encapsulation efficiency (Cullis and Hope, 2017; Oberli et al., 2017). However, nanoparticles generated with early fluidic devices based on macroscopic mixing techniques have often shown high polydispersity and poor reproducibility. For this reason, microfluidic chip devices have been recently developed for the synthesis of mRNA lipid-based nanoformulations (Cullis and Hope, 2017; Thomas et al., 2018). The use of microfluidic mixing devices can ensure a rapid mixing of the aqueous and organic phases, with a consequent fast increase of the polarity of the solution. The time required for mixing in the microfluidic mixer (tm) decreases with the flow velocity (U), according to the following formula: tmix ~ λ/[U ln(Ul/D)], where λ and l are parameters determined by the geometry of the microfluidic chip and D is the diffusion coefficient (Reichmuth et al., 2016; Cullis and Hope, 2017).

The effect of flow rate on the size and polydispersity of lipid-based nanoparticles has been investigated, and previously published studies report that size and polydispersity decrease with an increasing flow rate (Belliveau et al., 2012; Reichmuth et al., 2016). However, an increment in flow rate above 2 ml/min has no effect on nanoparticle's size (Belliveau et al., 2012; Reichmuth et al., 2016). Additionally, Zhigaltsev et al. tested the influence of aqueous/ethanol flow rate ratios on size and polydispersity, and identified a flow rate ratio of 3:1 as the limit value at which smaller and more uniform nanoparticles can be obtained (Zhigaltsev et al., 2012). All together, these findings suggest that an aqueous flow rate of 1.5 ml/min and an ethanol flow rate of 0.5 ml/min represent the best conditions to ensure the synthesis of monodisperse limit-sized nanoparticles.

The microfluidic mixer approach represents an innovative synthesis strategy that offers several advantages compare to other synthesis methods, allowing large-scale production of lipid-based nanoparticles with high encapsulation efficiency, small size and high monodispersity, and facilitates the manufacturing process of commercial mRNA drugs, according to GMP standards.

### mRNA to Deliver Different “Immunotherapeutic Messages”

mRNA-based gene therapy holds the promise to revolutionize the field of cancer immunotherapy by addressing current manufacturing limitations and offering novel therapeutic solutions.

The relative rapidity of its production is considered one of the central advantages of mRNA compared with other immunotherapeutic strategies. Indeed, the synthesis of mRNA-based treatments can be achieved within weeks using a cell-free and scalable process, once the sequence encoding the immunogene is available (Wadhwa et al., 2020).

Besides the manufacturing advantages, the use of mRNA technology can avoid any risk of genomic integration, since mRNA translation occurs in the cytosol and it is degraded naturally after gene expression (Granot and Peer, 2017; Guevara et al., 2019b). These characteristics indicate that mRNA-based immunotherapy has the potential to be safer than other strategies and is thus a promising immunotherapeutic platform.

Currently, mRNA constructs have been employed to express tumor associated antigens (TAA) and neoantingens for the development of therapeutic and prophylactic vaccines, for the *in-situ* production of potent monoclonal antibodies and for the engineering of CAR T cells (Kranz et al., 2016; Rybakova et al., 2019; Wilk et al., 2020). The application of mRNA for these strategies will be discuss in the next sections.

Additionally, a number of clinical trials are now examining the efficacy of nanoformulated mRNA cancer immunotherapies for different types of solid tumors and hematological malignancies (see Table 1).

**Table 1:** Clinical trials for formulated mRNA anti-cancer immunotherapies.

**Treatment**	**Phase**	**mRNA-encoded protein**	**Tumor**	**Identifier**
Vaccine	1	Four mRNAs encoding New York Esophageal Squamous Cell Carcinoma-1 (NY-ESO-1), Melanoma-associated antigen 3 (MAGE-A3), tyrosinase, and transmembrane phosphatase with tensin homology (TPTE)	Advanced malignant melanoma	NCT02410733
Vaccine	1	mRNA-4157 targeting 20 tumor-associated antigens (TAAs) that are specifically expressed by the patient's cancer cells	Resected solid tumors including melanoma, bladder carcinoma, and non-small-cell lung carcinoma (NSCLC), and in combination with pembrolizumab in patients with unreseactable solid tumors	NCT03313778
Immune modulator	1/2	mRNA-2416 encoding OX40 ligand (OX40L)	Alone or in combination with durvalumab for patients with solid tumors or lymphoma.	NCT03323398
Immune modulator	1	mRNA-2752 encoding OX40L, IL-23 and IL-36γ	Alone or in combination with duvalumab for patients with triple negative breast cancer, head and neck squamous cell carcinoma, non-hodgkin lymphoma, and urothelial cancer	NCT03739931

### mRNA General Structure

**Figure 6:**
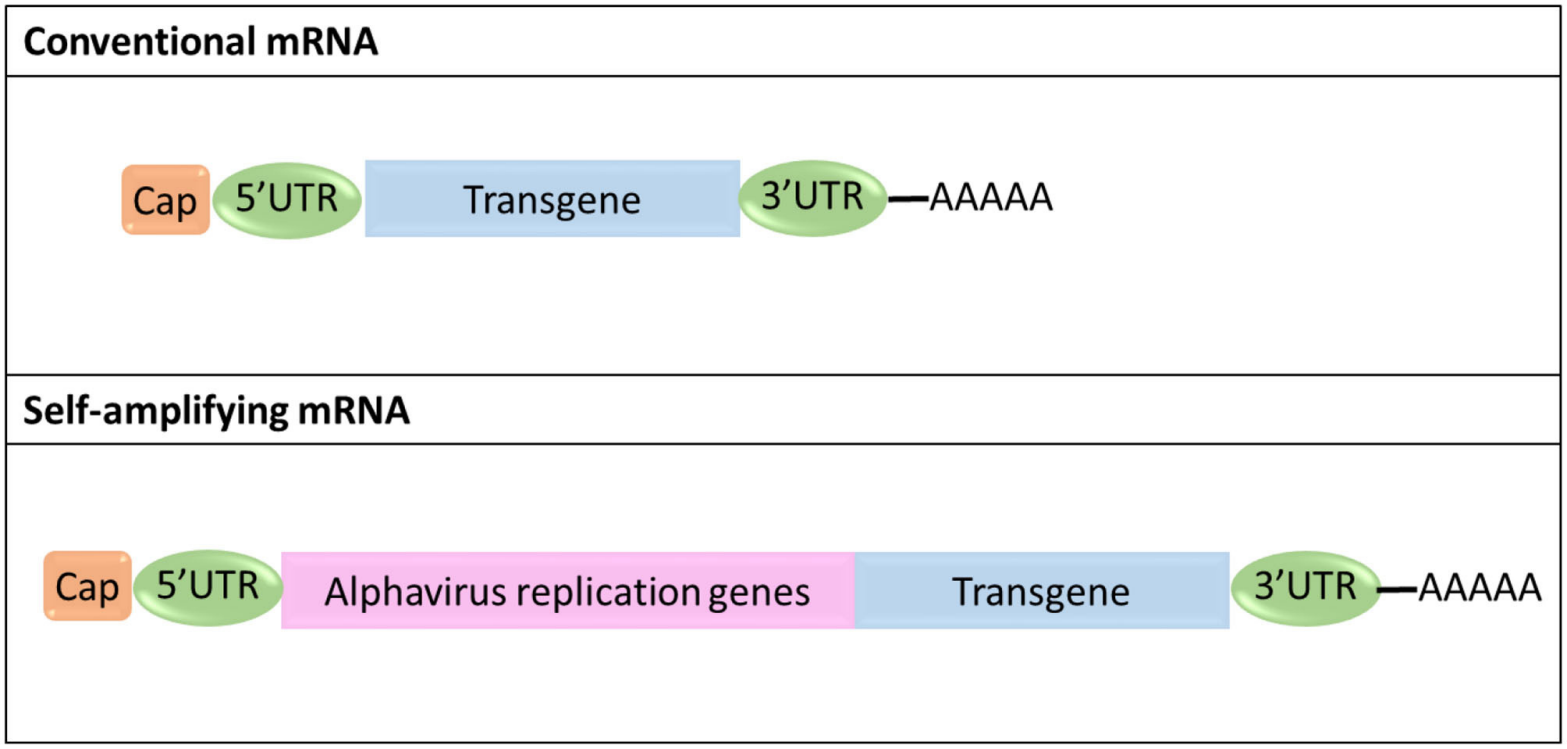
Schematic representation of the structural organization of conventional and self-amplifying mRNA.

Currently, two different types of mRNA platforms have been proposed for cancer immunotherapy, non-amplifying mRNA (conventional mRNA) with an open reading frame (ORF) flanked by 5′ and 3′ untranslated regions (UTRs), and self-amplifying mRNA (saRNA) derived from the positive-stranded alphavirus RNA genome (Figure 6) (Kowalski et al., 2019). In saRNA, genes encoding structural proteins are replaced by genes coding for proteins of therapeutic value, whereas viral genes containing the information for proteins forming the replication machinery are maintained (Kowalski et al., 2019).

Major advantages of conventional mRNA include its relatively small size compared to saRNA (~2–3 kb vs. ~10 kb), the absence of viral genes thus minimizing the risk of eliciting undesired immunogenic effects in the patient, its simple and scalable manufacturing procedures, and the fact that its sequence can be easily engineered to improve its therapeutic efficacy and minimize any adverse effects (Pardi et al., 2018; Kowalski et al., 2019). On the other hand, saRNA can produce multiple copies of itself, thereby achieving effective gene expression and protein translation with a much lower number of molecules compared to conventional mRNA (Kowalski et al., 2019).

Both types of mRNAs are synthesized in a cell-free system, using an *in vitro* transcription (IVT) method, which requires the generation of pDNA containing the sequence for a DNA-dependent RNA polymerase promoter (T7 or SP6), followed by the sequence corresponding to the mRNA construct (Weissman, 2014; Zhong et al., 2018; Kowalski et al., 2019). After enzymatic linearization, the pDNA can serve as a template for the transcription of mRNA using a DNA-dependent RNA polymerase. Once the transcription reaction is completed the pDNA is degraded by treatment with DNase. The addition of the 3′ poly(A) tail can be achieved during the transcription process or enzymatically after transcription via poly-A polymerase, while enzymatic addition of the 5′ cap can be carried out by using guanylyl transferase and 2′-O-methyltransferase to introduce a Cap 0 (N7MeGpppN) or Cap 1 (N7MeGpppN2′-OMe) structure, respectively (Weissman, 2014; Zhong et al., 2018; Kowalski et al., 2019).

mRNA sequence and its secondary structures can be potentially recognized by several innate immune receptors to promote the release of type I IFN, with a consequent inhibition of protein translation (De Beuckelaer et al., 2016). However, innate immune activation can be prevented by using modified mRNA, incorporating non-standard nucleotides such as pseudouridine (Ψ), 5-methylcytidine (5 mC), cap-1 structure and optimized codons, thus improving its translation efficiency (Holtkamp et al., 2006; Pardi et al., 2018).

The purity of the mRNA is a crucial factor that influences its performance. It has been shown that DNA-dependent RNA polymerases yield abortive initiation products, as well as double-stranded RNA resulting from self-complementary, which can lead to type I IFN and inflammatory cytokines production upon pattern recognition receptors recognition (Jackson et al., 2020). Karikó et al., in this regard, showed that the removal of impurities from synthetic mRNA by high-pressure liquid chromatography (HPLC) can minimize innate immune activation with a consequent significant increase of levels of expression of the reporter gene (Karikó et al., 2011).

### Lipid Nanoparticles for mRNA-Based Vaccines

The main objective of a therapeutic anti-cancer vaccine is to stimulate cell-mediated immune responses by targeting tumor antigens that are restricted or preferentially expressed in malignant cells (Pardi et al., 2018).

Adoptive transfer approaches, based on the administration of *ex-vivo* mRNA-transfected DCs, were the first form of mRNA-based vaccines to be proposed and clinically investigated (Baldin et al., 2020). Most of these clinical studies employed DCs generated from peripheral blood monocytes (Baldin et al., 2020). However, thanks to the recent advancements in separation techniques for primary DCs, the new generation of DC vaccines is focusing on the isolation of specific primary DC subsets, due to their superior immunostimulatory functions (Baldin et al., 2020).

Despite DC vaccines have shown encouraging outcomes in preclinical studies, their clinical efficacy remains limited. Additionally, there are multiple technical challenges associated with their manufacturing procedures (Farkona et al., 2016; Baldin et al., 2020). For instance, a large amount of patient's peripheral blood needs to be collected for the isolation and generation of DCs. This is a key issue when dealing with oncological patients, given that the cytotoxic effects of chemotherapy may further reduce the number of DCs and monocytes in the peripheral blood (Farkona et al., 2016; Baldin et al., 2020).

In the second decade of the twenty first century, direct *in vivo* transfection of DCs with a tumor antigen-encoding mRNA, appeared as a valid strategy to overcome limitations faced with the development of DC vaccines (Baldin et al., 2020). Since then, the field has been rapidly expanding, leading to the development of mRNA-based vaccines that are currently in clinical trials, and to the establishment of biotech companies across the globe focusing on mRNA technologies for cancer immunotherapy.

It is known that the efficacy of DC vaccines is strongly influenced by the efficiency of DCs to migrate toward secondary lymphoid organs after their administration to patients. However, *ex-vivo* activated DCs often fail to reach the lymph nodes and their functionality is affected by tolerogenic signals, impairing the ability of DCs to efficiently prime tumor-specific cytotoxic T cells (CTLs) (Turnis and Rooney, 2010; Farkona et al., 2016; Baldin et al., 2020). The release of the antigen directly into secondary lymphoid organs offers the possibility to reduce the risk that mRNA-transfected mature DCs receive inactivating signals before that they can encounter and present the antigen to naïve T cells (Kreiter et al., 2010). In addition, this kind of approaches can easily bypass all the technical limitations associated with the preparation of DC vaccines, since the isolation or generation of DCs is not necessary. First efforts in the delivery of antigen-mRNA demonstrated that local injection of naked mRNA into lymph nodes could promote antigen-specific anti-tumor immunity (Kreiter et al., 2010). However, its efficacy was negatively affected by the low uptake of naked mRNA by local DCs and the incapacity of free mRNA to escape from the endosomal compartment and reach the cytosol of the cells following its endocytosis. All these issues, together with the concerns related to the feasibility of direct intranodal injection of mRNA, has promoted the development of non-viral vectors opportunely designed for mRNA vaccine delivery.

Different types of nanoparticles have been proposed for mRNA delivery, but lipid-based nanoparticles have shown the most promising results and currently represent the gold standard for precise *in vivo* delivery of mRNA into immune cells (Kranz et al., 2016; Oberli et al., 2017; Persano et al., 2017; Miao et al., 2020). A proof-of-concept study using this approach was reported by Kranz et al. in 2016, demonstrating that DCs can be passively targeted *in vivo* using intravenously injected mRNA-lipoplexes based on DOTMA/DOPE or DOTAP/DOPE formulations by optimizing the mRNA/cationic lipid ratio, obtaining nanoparticles with a negative net charge (Kranz et al., 2016). The loading of TAA-mRNA onto the lipoplex nanostructure efficiently protected the mRNA from extracellular ribonucleases and enhanced its uptake and expression by different DC subsets and macrophages in various lymphoid organs. Finally, the authors showed that mRNA-lipoplex vaccine can induce both a type-I-IFN-mediated innate immune response as well as a potent adaptive response, resulting in a strong tumor growth inhibition.

After this study reported the successful application of lipid-based non-viral vectors for anticancer mRNA-based vaccines, other types of lipid-based nanostructures have been successfully employed, such as lipid nanoparticles and hybrid lipid-polymer formulations. For instance, Oberli et al. proposed a lipid nanoparticle-based formulation for the *in vivo* delivery of mRNA vaccines into APCs (Oberli et al., 2017). The efficacy of the vaccine was tested in a B16F10 melanoma murine model, detecting a strong anti-tumor cell-mediated immune response after a single dose. Treatment of B16F10 melanoma tumors with the nanovaccine containing mRNA coding for TAAs (gp100 or TRP-2) resulted in a decrease of the tumor volume and the prolongation of survival of treated mice. Likewise, Persano et al. reported the development of a hybrid lipid-polymer nanoformulation, consisting of poly-(β-amino ester) polymer/mRNA core coated with 1,2-dioleoyl-sn-glycero-3-ethylphosphocholine (EDOPC)/DOPE/1,2-distearoyl-sn-glycero-3-phosphorylethanolamine (DSPE)-PEG(2000), for mRNA vaccine delivery (Persano et al., 2017). This hybrid nanostructure was efficiently internalized by DCs via micropinocytosis and promoted their maturation through a mechanism that involves innate immunity activation by Toll-like receptor 7/8 signaling (Persano et al., 2017). The vaccination of mice bearing lung metastatic B16-OVA tumors with OVA-mRNA/lipopolyplex resulted in a significant reduction in the number of lung metastases (Persano et al., 2017).

Recently, mRNA-based nanovaccines have been explored for the development of a new class of anticancer vaccine, referred as personalized vaccines that are based on tumor neoantigens deriving from non-synonymous mutations occurring in cancer cells. In this direction, mRNA-based nanovaccines are attracting a growing interest, as they hold the unique potential to facilitate the co-release of multiple neoantigens by incorporating different epitope sequences within the same molecule. Kreiter et al. have provided evidences that neoantigens are immunogenic when delivered via mRNA platforms and currently several clinical trials are ongoing testing mRNA encoding for neoantigens for the treatment of various solid tumors, including NSCLC, colorectal and pancreatic cancer (Kreiter et al., 2010; Kowalski et al., 2019).

A deeper understanding of the mechanisms involved in the activation and setting up of immune responses has helped to determine the crucial role that a specific immune adjuvants included in a vaccine formulation can play in determining the therapeutic outcome of a vaccine in patients. Indeed, even if non-modified mRNA has well-recognized immune adjuvanting properties associated to its ability to interact with innate immune receptors, it is also true that mRNA vaccines can benefit from their combination with immune adjuvants directed to re-modulate the immunosuppressive microenvironment or to provide additional signals that can reinvigorate vaccine-induced immune responses (Verbeke et al., 2017; Guevara et al., 2019a). This is particularly relevant for oncological patients, as they tend to have a compromised immune system and may involve elderly adults that display features of immunosenescence (Crooke et al., 2019). In this regard, lipid nanoformulations have shown to efficiently assist in the co-delivery of antigen-mRNA with immune adjuvants, promoting the development of potent and effective anti-cancer vaccines. Haabeth et al., for instance, demonstrated that intratumoral injection of charge-altering releasable transporters (CART)-mRNA complexes resulted in high expression of the transduced transgene, with an efficient transfection of dendritic cells, macrophages, and T cells at the injection site (Haabeth et al., 2019). The co-delivery of OX40L-, CD80-, and CD86-encoding mRNAs resulted in a localized upregulation of pro-inflammatory cytokines, robust T cells priming, and migration of immune cells toward the draining lymph node or to distant tumors. This therapeutic approach significantly inhibited tumor growth and promoted tumor eradication in two different murine tumor models.

The field of mRNA-based nanovaccine is rapidly progressing and it currently represents the most advanced application of mRNA technology with multiple clinical trials ongoing in different tumor settings, including melanoma, bladder carcinoma and NSCLC (see Table 1).

The first successful application of a therapeutic nanovaccine based on multiple mRNAs encoding four tumor antigens (NY-ESO-1, MAGE-A3, tyrosinase, and TPTE) was tested in advanced malignant melanoma patients (NCT02410733, phase I). The study showed that all patients developed de novo T cell responses against the administrated tumor antigens. Additionally, this multiple mRNA nanovaccine was successfully evaluated in combination with checkpoint inhibitor in patients with unresectable solid tumors (Kranz et al., 2016).

These early positive indications highlight the potential of mRNA nanovaccines, and provide evidences that this strategy may be at the point of development to be incorporated into clinical practice. However, the enormous therapeutic potential of mRNA is still limited by the need for improved targeting and transfection efficiency of lipid-based mRNA delivery systems.

### Lipid Nanoparticles for the Delivery of mRNA Coding for Monoclonal Antibodies

Monoclonal antibodies represent one of the most studied class of cancer immunotherapy and they have received clinical approval for the treatment of an increasing number of human malignancies (Hoecke and Roose, 2019). Antibody-based cancer immunotherapy has been applied to target specific proteins express on tumor cells and immune cells or molecules released into the extracellular environment. Depending on the protein they are targeting, antibodies have different mechanisms of action and effects (Suzuki et al., 2015).

Cancer treatment based on monoclonal antibodies targeting immune checkpoints is largely considered the most promising area of cancer immunotherapy currently in development (Park et al., 2018). Several types of immune checkpoints monoclonal antibodies have been discovered in the last years and some of them, such as anti-programmed cell death 1 (PD-1)/programmed cell death-ligand 1 (PD-L1) and cytotoxic T-lymphocyte-associated protein 4 (CTLA-4) inhibitors, have already received approval for clinical use while many others are under clinical trials (Park et al., 2018).

Although monoclonal antibodies have generally exhibited great therapeutic efficiency in the context of diverse solid tumors, their use is associated with several disadvantages that limit their extensive application in the clinic. These challenges are mainly related to the manufacturing process of antibodies, which requires the use of engineered mammalian cells followed by complex and time-consuming procedures in order to obtain an antibody completely free from cell culture supernatant, viruses and other potential contaminants (Hoecke and Roose, 2019; Schlake et al., 2019). Furthermore, monoclonal antibodies are characterized by a wide variety of post-translational modifications, which can strongly impact their therapeutic properties (Hoecke and Roose, 2019; Schlake et al., 2019). Therefore, after synthesis and purification, the quality of antibodies needs to be assess using many expensive analytical techniques (Hoecke and Roose, 2019; Schlake et al., 2019). All these challenges render antibody-based therapies poorly affordable.

In the recent years, mRNA technology has emerged has an elegant solution to circumvent the limitations associated with the preparation of antibody-based drugs (see Table 1) (Hoecke and Roose, 2019; Schlake et al., 2019). With this innovative approach, by administrating the antibody-encoding mRNA directly to patients, it is possible to achieve *in situ* production of the therapeutic product, overcoming all the problems associated to its synthesis and purification (Hoecke and Roose, 2019; Schlake et al., 2019).

A proof of the feasibility of using mRNA as a platform for antibody-based immunotherapy was reported by Pardi et al. In this work, modified mRNAs encoding both the light and heavy chains of a neutralizing antibody direct against HIV-1 (VRC01), were co-encapsulated into lipid nanoparticles and intravenously administrated (Pardi et al., 2017). Passive vaccination with mRNAs-loaded nanoparticles led a robust antibody expression in the liver, resulting in an effective prophylactic response in a HIV-1 murine model (Pardi et al., 2017). Using a similar strategy, Stadler et al. reported a new class of drug that employs a modified mRNA formulated into lipid nanoparticles to promote *in situ* production of bispecific antibodies termed RiboMABs. RiboMAB targeting CD3 and TAAs link T cells to cancer cells, enhancing the anti-tumor activity of effector cells (Stadler et al., 2017).

A single dose of mRNA-loaded nanoparticles, administrated intravenously, was sufficient to promote rapid production of bispecific antibodies and their secretion into circulation. Treatment with RiboMAB completely eliminated the tumor and remarkably, in a control experiment, to achieve a similar degree of tumor eradication the recombinant bispecific antibody had to be administered three times (Stadler et al., 2017).

All the above-mentioned studies have delivered the mRNA intravenously, exploiting liver cells as a sort of bioreactor to translate the mRNA and release antibodies systemically. In contrast, Tiwari et al. achieved local expression of antibodies directed against the respiratory syncytial virus (RSV) by delivering formulated mRNA encoding antibody in the lungs via intratracheal aerosols (Tiwari et al., 2018). The authors showed that by using this delivery approach, up to 45% of the lung cells expressed the antibody, leading to a significant reduction of RSV infection in challenged mice (Tiwari et al., 2018).

These studies demonstrate the potential of mRNA-based lipid nanovectors as platforms for the *in situ* production of antibodies, and how their use may revolutionize the field. Particularly exciting is the fact that this technology may reduce the cost and the number of doses currently require for treatments with recombinant monoclonal antibody-based therapies, thus rendering them more accessible to a larger portion of patients.

### Lipid Nanoparticles to Harness mRNA Therapeutic Potential for CAR T Cell Therapy

CAR T-cell therapy represents the most advanced personalized cancer immunotherapy and has received approval from the FDA and the European Medicine Agency (EMA) for its clinical implementation in the context of hematological cancers, including acute lymphoblastic leukemia and diffuse large B-cell lymphoma (Mohanty et al., 2019; Vitale and Strati, 2020). Thus, CAR T cell therapy is one of the first successful examples of cell engineering and personalized adoptive cell transfer immunotherapy to become available in clinic.

In this strategy, T cells are isolated from the patient and genetically modified to introduce a chimeric antigen receptor that binds a tumor protein that is express uniquely or mostly by the target malignant cells. Afterward, CAR T cells are expanded and re-infused into patients to attack and destroy chemotherapy-resistant cancer cells (Jackson et al., 2016).

Although this therapeutic approach is currently restricted to the treatment of non-solid tumors, thanks to the recent advancement in the field and the introduction of novel technologies, the scientific community is largely sure that in the next future it would be possible to extend this treatment regime to the treatment of solid tumors.

Despite its tremendous potential, previous studies with CAR cell therapy have pointed out several limitations concerning safety issues, complex manufacturing procedures and high costs, that can hinder the wide application of this technology (Hartmann et al., 2017; Zhao et al., 2018).

Regarding the collateral effects, they have been mostly associated to unwanted immunological immune responses that can lead to macrophage activation syndrome, neurotoxicity and cytokine release syndrome. Concerns have been also raised regarding the use of viral vectors for transducing T cells with CAR sequences, particularly due to their immunogenicity and the potential risk of insertional mutagenesis, besides their limited size insert capacity (Hartmann et al., 2017; Zhao et al., 2018).

While immunological toxicity may be mitigated by treatment with anti-IL-6 receptor antibodies, manufacturing challenges remain unsolved, justifying the need for novel lymphocyte transfection strategies for the development of safer and more accessible CAR cell therapies (Brudno and Kochenderfer, 2016).

Recently, mRNA technology has emerged as a potential solution to overcome these challenges. Indeed, mRNA allows the transient expression of CAR, since mRNA molecules are subject to decay after translation, thus preventing any risk of genomic vector integration (Wiesinger et al., 2019). Furthermore, the structure of the mRNA can be easily customized with specific sequences or modifications to maximize transfection and translation.

Currently, electroporation is standardly employed in clinical practice to deliver mRNA encoding CAR into T cells. However, electroporation has several disadvantages that can strongly affect the quality of the CAR T cells produced (Billingsley et al., 2020). Indeed, the application of pulsed electric fields can irreversibly compromise the cell plasma membrane's integrity. All this can result in low viability, aberrant gene expression profile and reduced transgene expression in the surviving transfected cells. At the end of the last decade, non-viral delivery systems have been explored as an alternative approach for lymphocytes' transfection (Billingsley et al., 2020). In particular, ionizable lipid nanoparticle delivery platforms have showed outstanding efficacy in preclinical studies (see Table 2). In line with the above, Billingsley et al. recently reported the development of ionizable lipid nanoparticles for *ex vivo* mRNA delivery into human T cells. The designed nanovector was exploited to achieve CAR-encoding mRNA delivery into primary human T cells to produce functional CAR T cells with enhanced tumor killing activity (Billingsley et al., 2020).

**Table 2:** Overview of formulated mRNA strategies for monoclonal antibody and CAR cell therapies.

**Type of immunotherapy**	**Nanocarrier composition**	***Ex-vivo* or *in vivo* transfection**	**Transgene**	**Tumor**	**References**
Monoclonal antibody	C14-4/DOPE/Chol/PEG-lipid (35:16:46.5:2.5 mol/mol)	*In vivo*	Anti-HER2 antibody	MDA-MB-231 cells (Breast cancer)	Rybakova et al., 2019
	Polymer/lipid formulation	*In vivo*	CLDN6 × CD3 bispecific antibody	OV-90 cells (Ovarian cancer)	Stadler et al., 2017
	L319/DSPC/chol/PEG-DMG (50:10:38.5:1.5 mol/mol)	*In vivo*	Anti-CD20 antibody (Rituximab)	Raji cells (Burkitt's lymphoma)	Thran et al., 2017
CAR cell therapy	C14-4/DOPE/Chol/PEG-lipid (35:16:46.5:2.5 mol/mol)	*Ex-vivo*	CD19	Nalm6 cells (Acute lymphoblastic leukemia)	Billingsley et al., 2020
	CART synthetic lipid-based nanoparticles	*Ex-vivo* and *in vivo*	GFP and Luciferase	ND	McKinlay et al., 2018
	CART synthetic lipid-based nanoparticles	*Ex-vivo*	CD19	Nalm6 cells (Acute lymphoblastic leukemia)	Wilk et al., 2020

Interestingly, recent studies have highlighted the great potential of mRNA-based lipid nanoformulations to deliver genetic material to the target cells directly *in vivo*, thus avoiding the complications associated with the *ex-vivo* manipulation of T cells. This kind of strategies can offer the unprecedented possibility to easily transfect T cells using a practical and broadly applicable approach.

## Conclusions and Future Prospective

Synthetic mRNA has gained a growing interest as a therapeutic molecule for preventing or treating multiple malignancies or non-oncological diseases. The idea to use mRNA as therapeutic molecule was born due to the number of benefits that its implementation can offer over traditional treatments.

As mentioned in the previous sections, oppositely to pDNA-based gene therapy, mRNA holds a superior safety profile, given that it does not need to reach nucleus to exert its function, thus avoiding any risk of genomic integration. In addition, mRNA expression is time-restricted and can be tightly regulated. Most importantly, mRNA-based therapy can allow a rapid and affordable manufacturing of therapeutics as its synthesis is achieved using cell-free systems.

Despite this, mRNA is chemically unstable and susceptible to hydrolysis catalyzed by nucleases. The great structural fragility of mRNA limited its use as therapeutic agent in the past. Recent advances in non-viral delivery systems and the development of novel effective transfecting nanomaterials have provided solutions to these challenges.

Nowadays, lipid-based nanoformulations represent the most advanced and widely employed delivery system for the development of mRNA-based therapies. With several mRNA-based anti-cancer treatments currently in preclinical and clinical studies, it is evident that cancer immunotherapy is the field in which mRNA-based technology can better exert its enormous therapeutic power.

The application of lipid-based nanovectors has enabled the integration of mRNA-based technology in many pre-existing anti-cancer immunotherapeutic approaches, such as therapeutic vaccines, monoclonal antibodies and CAR cell therapy.

However, further research is needed to clarify the reasons behind the low mRNA transfection efficiency of non-viral vectors, especially in those cells that are considered hard to transfect, such as lymphocytes and monocytes. Recent published works have shown how the co-formulation of mRNA with drugs known to affect that endocytic pathway, can significantly enhance gene delivery (Patel et al., 2017; Kon et al., 2020). Modulating intracellular transport mechanisms for mRNA internalization and endosomal escape will potentially lead to the development of next generation of drug delivery systems by enabling high transfection efficiency with limited toxicity.

Additionally, a deeper understanding of key parameters, such as hydrophobicity and fusogenicity of the formulation, which strongly dictate the transfection efficiency of the formulation, and how they can be modulated by varying the lipidic composition or through the introduction of novel lipids, will further enable the development of improved formulations.

Finally, combination with other cancer treatments, including chemotherapy and radiotherapy represents a promising way to further potentiate the therapeutic efficacy of mRNA-based strategies.

## Author Contributions

SP and MG designed and wrote the manuscript. FP contributed to the writing and to revising the manuscript. All authors contributed to the article and approved the submitted version.

## Conflict of Interest

The authors declare that the research was conducted in the absence of any commercial or financial relationships that could be construed as a potential conflict of interest.
